# Characterization of Two *Zygnema* Strains (*Zygnema circumcarinatum* SAG 698-1a and SAG 698-1b) and a Rapid Method to Estimate Nuclear Genome Size of Zygnematophycean Green Algae

**DOI:** 10.3389/fpls.2021.610381

**Published:** 2021-02-10

**Authors:** Xuehuan Feng, Andreas Holzinger, Charlotte Permann, Dirk Anderson, Yanbin Yin

**Affiliations:** ^1^Department of Food Science and Technology, Nebraska Food for Health Center, University of Nebraska-Lincoln, Lincoln, NE, United States; ^2^Department of Botany, University of Innsbruck, Innsbruck, Austria; ^3^Center for Biotechnology, University of Nebraska-Lincoln, Lincoln, NE, United States

**Keywords:** DAPI staining, electron transport rate, flow cytometry, nuclear genome size estimation, mechanic chopping, xanthophyll cycle pigments, *Zygnema circumcarinatum*

## Abstract

Zygnematophyceae green algae (ZGA) have been shown to be the closest relatives of land plants. Three nuclear genomes (*Spirogloea muscicola*, *Mesotaenium endlicherianum*, and *Penium margaritaceum*) of ZGA have been recently published, and more genomes are underway. Here we analyzed two *Zygnema circumcarinatum* strains SAG 698-1a (mating +) and SAG 698-1b (mating −) and found distinct cell sizes and other morphological differences. The molecular identities of the two strains were further investigated by sequencing their 18S rRNA, *psaA* and *rbcL* genes. These marker genes of SAG 698-1a were surprisingly much more similar to *Z. cylindricum* (SAG 698-2) than to SAG 698-1b. Phylogenies of these marker genes also showed that SAG 698-1a and SAG 698-1b were well separated into two different *Zygnema* clades, where SAG 698-1a was clustered with *Z. cylindricum*, while SAG 698-1b was clustered with *Z. tunetanum*. Additionally, physiological parameters like ETR_max_ values differed between SAG 698-1a and SAG 698-1b after 2 months of cultivation. The de-epoxidation state (DEPS) of the xanthophyll cycle pigments also showed significant differences. Surprisingly, the two strains could not conjugate, and significantly differed in the thickness of the mucilage layer. Additionally, ZGA cell walls are highly enriched with sticky and acidic polysaccharides, and therefore the widely used plant nuclear extraction protocols do not work well in ZGA. Here, we also report a fast and simple method, by mechanical chopping, for efficient nuclear extraction in the two SAG strains. More importantly, the extracted nuclei were further used for nuclear genome size estimation of the two SAG strains by flow cytometry (FC). To confirm the FC result, we have also used other experimental methods for nuclear genome size estimation of the two strains. Interestingly, the two strains were found to have very distinct nuclear genome sizes (313.2 ± 2.0 Mb in SAG 698-1a vs. 63.5 ± 0.5 Mb in SAG 698-1b). Our multiple lines of evidence strongly indicate that SAG 698-1a possibly had been confused with SAG 698-2 prior to 2005, and most likely represents *Z. cylindricum* or a closely related species.

## Introduction

About 450 million years ago, some ancient charophycean green algae (CGA) emerged from the aquatic habitats to colonize terrestrial environments (Delwiche and Cooper, 2015). Modern CGA consist of six classes, which form two major clades, the ZCC-clade (Zygnematophyceae, Charophyceae, and Coleochaetophyceae), and the KCM-clade (Klebsormidiophyceae, Chlorokybophyceae, and Mesostigmatophyceae) (de Vries et al., 2016). Compared to KCM, ZCC are phylogenetically closer to Embryophyta (land plants), and the class Zygnematophyceae has been shown to be the sister lineage to land plants (Wodniok et al., 2011; Timme et al., 2012). Therefore, sequencing the genomes of Zygnematophycean green algae (ZGA) will contribute to the understanding of the origin and early evolution of land plants. Indeed, the first two ZGA genomes have been published in 2019 from *Spirogloea muscicola* and *Mesotaenium endlicherianum*, revealing that horizontal gene transfer from soil bacteria had played a critical role for ancient plant terrestrialization and stress resistance (Cheng et al., 2019). In addition, the *Penium margaritaceum* genome has been more recently described (Jiao et al., 2020), and more nuclear genomes are on their way.

We have been working on genome sequencing of four strains of the first filamentous ZGA, *Zygnema circumcarinatum*: UTEX 1559 (mating +), UTEX 1560 (mating −), SAG 698-1a (mating +), and SAG 698-1b (mating −). We (Fitzek et al., 2019; Orton et al., 2020) and others (de Vries et al., 2018) have also recently reported the transcriptome and organellar genomes of *Z. circumcarinatum*. The UTEX 1559 strain resulted from a spontaneous mutation of LB42 (IUCC 42, former Indiana University Culture Collection, now UTEX 42) isolated by Gauch (1966). The UTEX 1560 is a spontaneous mutation of LB43 (IUCC 43).

However, there have already been some doubts about the species identity of SAG 698-1a (Stancheva et al., 2012) compared to other strains of *Z. circumcarinatum*. These authors found that the sequences of *Z. circumcarinatum* from the MZCH (Microalgae and Zygnematophyceae collection of Hamburg) and UTEX strains are markedly different from the *rbcL* gene derived from the whole chloroplast genome of SAG 698-1a (Turmel et al., 2005). Moreover, in the *rbcL* phylogeny the position of SAG 698-1a (Stancheva et al., 2012) is different from that of UTEX 42 as reported in Hall et al. (2008) and Pichrtová et al. (2018). They speculated that the published chloroplast genome of strain SAG 698-1a is not that of *Z. circumcarinatum*, but rather that of some other species of *Zygnema* (Stancheva et al., 2012). Recently the chloroplast genome of UTEX 1559 was sequenced (Orton et al., 2020), sharing only 85.69% sequence identity with that of SAG 698-1a sequenced in Turmel et al. (2005). This further suggested that the published chloroplast genome of SAG 698-1a is not that of *Z. circumcarinatum* (Orton et al., 2020). Therefore, the primary goal of this study is to perform a comprehensive characterization of SAG 698-1a and the corresponding mating – strain SAG 698-1b, by phylogenetic, morphological, morphometric, physiological, and sequence analyses.

Regarding its physiology and ecology, *Z. circumcarinatum* belongs to a group of conjugating algae with unbranched filaments. Previous studies have shown that *Z. circumcarinatum* was isogamous and the conjugation was scalariform, where gametes of both mating types were released to the medium and formed brownish orange zygospores (Gauch, 1966; Miller, 1973). Its cells contain on average two star-shaped chloroplasts, where a single nucleus is located in between (Gauch, 1966). *Z. circumcarinatum* inhabits shallow freshwater and watery soil environments such as near shores of lakes and rivers. This species has recently been used for several transcriptomic studies (Rippin et al., 2017; de Vries et al., 2018). Different species of *Zygnema* can be found all over the world, including Arctic and Antarctic areas (Holzinger et al., 2009; Pichrtová et al., 2018; Rippin et al., 2019), probably because they have evolved genes to tolerate stresses from extreme environments, such as cold and desiccation (Rippin et al., 2017; de Vries et al., 2018).

To sequence the nuclear genomes of *Z. circumcarinatum*, we have explored new experimental protocols to extract nuclear DNAs of high molecular weight. The reason is that all the previous methods had failed in *Z. circumcarinatum*, due to the excessive amount of mucilage present in the cultures, which were extremely difficult to remove using traditional methods. Recent research in different ZGA has reported that on the surface of their cell walls sticky mucilage is present (Herburger et al., 2019; Palacio-López et al., 2019; Jiao et al., 2020). These polysaccharides include large amounts of homogalacturonan pectins (polymers of galacturonic acid) and arabinogalactan proteins (AGPs, with polymers of galactose and arabinose), which are known to be sticky to hold algal filaments together forming mats and retain water against dehydration to adapt to semi-terrestrial environments.

To experimentally estimate the nuclear genome size of *Z. circumcarinatum* that can guide the genome assembly, we had to explore nuclei extraction protocols so as to use flow cytometry (FC). FC is a rapid and powerful technology, widely used in cell sorting and also for analyzing DNA content and ploidy levels in plants (Galbraith et al., 1983; Doležel and Bartoš, 2005; Doležel et al., 2007; Ibrahim and van den Engh, 2007). To conduct FC analysis, one has to obtain high quality nuclei samples. In 1983, (Galbraith et al., 1983) developed a plant nuclei isolation method for cell cycle analysis with FC. Since then, a variety of buffers and protocols, based on Galbraith’s method, have been developed to extract nuclei and applied to measurements of plant DNA content (Doležel et al., 2007). Previous reports had described nuclear genome size estimations using FC in red algae (Hong et al., 2016), brown algae (Phillips et al., 2011) and green algae (Mazalová et al., 2011). However, the methods used in these reports suffered from chromatin structure change leading to low accuracy of DNA content measurements (Doležel et al., 2007). In particular, for ZGA, the excessive mucilaginous materials can further decrease the accuracy of FC (Loureiro et al., 2006; Bennett et al., 2008; Cires et al., 2011; Wang et al., 2015). Therefore, the secondary goal of this study is to develop an efficient nuclei extraction protocol for nuclear genome size estimation.

In this paper, we aim to describe an improved method for nuclei extraction, modified from the original method of Galbraith et al. (1983). This method has been successfully used in our *Z. circumcarinatum* genome sequencing project and can be generalized and applied to other extracellular polysaccharide mucilage-rich ZGA. More importantly, for our primary goal we also present results from morphological, morphometrical and physiological investigations, and a nuclear genome size estimation of SAG 698-1a and SAG 698-1b, and conclude that the SAG 698-1a strain is unlikely the mating + strain of *Z. circumcarinatum*. Instead SAG 698-1a must have been mis-labeled and should represent a different species than *Z. circumcarinatum*. Overall, the two *Zygnema* strains are characterized as two different species. The nuclear genome size estimation result also informs their genome assembly and annotation that are currently underway.

## Materials and Methods

### Algae Culturing

Axenic *Z. circumcarinatum* SAG 698-1a and SAG 698-1b cultures were obtained from the Culture Collection of Algae at the University of Göttingen, Germany (SAG) in 2017, and grown in Plant Growth Chambers (Conviron PGR15) with ∼50 μmol photons m^–2^ s^–1^. The filaments were grown in liquid Bold’s Basal Medium (BBM) or modified BBM (Fitzek et al., 2019; Orton et al., 2020) for less than 2 weeks on a shaker platform at 110 rpm in Precision Plant Growth Chamber (Thermo Fisher Scientific, United States) (16/8 of light/dark cycle, 20°C). More details can be found in our previous papers (Fitzek et al., 2019; Orton et al., 2020). Independently, these two cultures were obtained from SAG in 2019 again and subcultured on solidified 1.5% agar containing BBM with added vitamins and incubated in a Percival incubator at ∼40 μmol photons m^–2^ s^–1^ at a 16/8 light/dark cycle at 20°C during light and 15°C during darkness. From each subculture three independent biological replicates were made, and cultivated for up to 4 months.

### DNA Sequencing of Marker Genes

Eight single filaments of SAG 698-1a with obvious morphological differences were picked under a dissecting microscope with the micromanipulation method described in Gauch (1966). Each single filament was transferred onto a MBBM 1% agar plate and cultivated for 3 months under the standard algae culture condition. The algae were harvested using a vacuum filtration with Whatman #2 paper (GE Healthcare 47 mm), washed with distilled water for three times, frozen in liquid nitrogen and stored at −80°C. The frozen algae were lyophilized overnight before DNA extraction. DNA was extracted from the eight algal clones with DNeasy PowerPlant Pro Kit (Qiagen, Germany). The genomic DNA was then used as the template for PCR amplification of 18S rRNA, *psaA*, and *rbcL* genes with GoTaq^®^ G2 Flexi DNA Polymerase (Promega, United States). The primers used were listed in Supplementary Table 1. The PCR products were purified with GeneJET Gel Extraction Kit (Thermo Fisher Scientific, United States) and sequenced at Roy J. Carver Biotechnology Center at University of Illinois at Urbana-Champaign using Sanger sequencing. The three genes of SAG 698-1b were also sequenced using the same method. As the sequences of 18S rRNA, *rbcL* and *psaA* of SAG 698-1a are identical to previously published SAG 698-1a RNA sequences in GenBank (see section “Results”), we did not submit them to NCBI. We did not submit the 18S rRNA sequence of SAG 698-1b to NCBI for the same reason. However, the sequences of SAG 698-1b *rbcL* and *psaA* were submitted to NCBI and received GenBank accession numbers (MW267923 and MW267924, respectively).

### Phylogenetic Analysis of Marker Genes

In addition to the sequences from SAG 698-1a and SAG 698-1b, more sequences of 18S rRNA, *rbcL*, and *psaA* from other *Zygnema* species were retrieved from GenBank. These sequences were aligned by MAFFT (Katoh et al., 2002). Phylogenetic trees were built by using RAxML (Stamatakis, 2014) with the “−f a” method and the PROTGAMMAJTT model. Bootstrap replicate trees were calculated 100 times to generate bootstrap support values.

### Cell Width and Length

One week old cultures of SAG 698-1a and SAG 698-1b were used for cell width and length measurement. The algal filaments were transferred onto glass slides and viewed under an Axio Imager 2 microscope (Carl Zeiss Microscopy, LLC). The determination of the cell width in relation to the culture age was performed on 0.5, 1–4 months old cultures on solidified media with a Zeiss Axiovert 200 M microscope under the control of a Zeiss Axiovision software (release 4.7). Images were captured with a Zeiss Axiocam HRm Rev.3 camera (Carl Zeiss, Jena, Germany). For each culture age, 20 randomly selected cells from three independent biological replicates were measured. Statistical evaluation of the size variation between the different culture ages were performed by a multifactor ANOVA (SPSS, 25.0, Macintosh) and values were considered significantly different when *p* < 0.01. The comparison between the different strains were performed with a Mann–Whitney-*U*-test (SPSS, 25.0, Macintosh). Confocal laser scanning microscopy was performed with a Zeiss Pascal LSM5 under control of ZEN 2009 SP2 software for determination of the chloroplast morphology.

### Mucilage Sheath and Chloroplast Number per Cell

The mucilage sheath width of these cells was measured after an inverse ink staining protocol with commercial Indian ink (Dr. Martin’s Bombay blue), which stained the background but left the mucilage layer unstained. The cells were investigated by a Zeiss Axiovert 200 M microscope. In total, 50 randomly selected cells were used for mucilage sheath width measurements. The average number of chloroplasts per cell was determined by an evaluation of 200 cells per strain. Statistic evaluations of these data were performed with a Mann–Whitney-*U*-test (SPSS, 25.0, Macintosh).

### Exponential Growth and Performance

Growth was determined to ensure that both strains were in the exponential growth phase as previously described (Pichler et al., 2020). The fluorescence *F*_o_ of 20 min dark-adapted cultures was measured with a pulse-amplitude modulated fluorometer PAM 2500 (Heinz Walz GmbH, Effeltrich, Germany) every 3–4 days up to 51 days. For each strain eight petri dishes were prepared, each containing three subsamples. The measurement of the fluorescence of each individual algal spot was repeated three times. This method is noninvasive as the fluorescence value was recorded outside the lid of a sealed petri dish. The end of the exponential growth phase was then determined from the graphs at approximately day 32 when the samples were harvested for HPLC analysis. The electron transport rate (ETR)- irradiance curves were determined with the same PAM 2500 as previously described (Herburger et al., 2015). Electron transport rates (ETRs) were calculated as: ETR = PAR (μmol photons m^–2^ s^–1^) ⋅ (*F*_m_′–*F*_t_)/*F*_m_′⋅ 0.5 (PS1/PS2 allocation factor) ⋅ 0.84 (incident light conversion efficiency in green plants). *F*_m_′ is the maximal chlorophyll fluorescence yield when photosystem II reaction centers are closed by a strong light pulse (saturation pulse). *F*_t_ is the continuously recorded fluorescence (in the actinic light-adapted state). Primary pigments were analyzed at the end of the exponential growth phase by a standard HPLC protocol (Holzinger et al., 2018) on an Agilent Technologies 1100 system (Waldbronn, Germany) with a DAD-detector set at 440 nm for carotenoids and 662 nm for chlorophyll a. The column was a LiChroCART (C18, 100 mm × 4.6 mm, 5 μm, 120 A; Agilent, Waldbronn, Germany) and a flow rate of 1 ml min^–1^ with solvent A (acetonitrile:methanol = 76:1) and solvent B (methanol:hexane 5:1). The determination of the de-epoxidation state (DEPS) of the xanthophyll cycle pigments was given by the ratio of antheraxanthin (A), violaxanthin (V), and zeaxanthin (Z) whereby: DEPS = (A + Z)/(V + A + Z). Pigment analysis was statistically performed with the Mann–Whitney-*U*-test and considered significant when *p* < 0.05 (SPSS, 25.0, Macintosh).

### Conjugation Test

Conjugation of SAG 698-1a and SAG 698-1b was tested following the method described in Gauch (1966). Specifically, 1-month old filaments of the two strains were mixed and transferred onto glass slides containing C-medium (Gauch, 1966). The slide was then covered with cover glass and put into a petri dish plate with 1 ml of water in the bottom. The plate was sealed with parafilm and placed into a plant growth chamber (Thermo Fisher Scientific, United States). In addition, plain agar (distilled water with 1% agar) (Gauch, 1966) and 0.1 diluted medium (Miller, 1973) were also exploited to induce conjugation with 1-month old algal cultures using the aforementioned method. The culturing condition was: ∼50 μmol photons m^–2^ s^–1^, 16/8 of light/dark cycle, 20°C. Conjugation was visually inspected using an Axio Imager 2 microscope (Carl Zeiss Microscopy, LLC) every 24 h for 7 days after the two strains were mixed.

### Nuclei Isolation

Young cultures (2 weeks) were harvested using a vacuum filtration with Whatman #2 papers (GE Healthcare 47 mm), and washed with water for three times. Nuclei isolation was prepared according to Galbraith et al. (1983) with modifications, described as follows. ∼50 mg of 2 weeks old algal filaments were transferred to plastic petri dishes and chopped with a single edge razor blade for 5 min. Then, the chopped algal filaments were transferred to a 1.5 ml Eppendorf tube and immediately put on ice. Then, 0.5 ml of nucleus extraction buffer (CyStain PI Absolute P kit, Sysmex) were added and gently mixed. The samples were filtered through a 20 μm CellTrics filter (Sysmex) into a collection tube cooled over ice. Samples were then centrifuged with 600 × *g* at 4°C for 10 min and the supernatant decanted. Pellets were washed with 1 ml of nucleus extraction buffer, and centrifuged again with 600 × *g* at 4°C for 10 min. The washing step was repeated two more times.

*Arabidopsis thaliana* nuclei were used as control for FC (see below). Three young leaves were taken and transferred into a 1.5 ml Eppendorf tube. Then, 0.5 ml of nucleus extraction buffer was added. The leaves were chopped into very small pieces with spatula for 1 min. The extraction mixture was filtered through a 20 μm CellTrics filter. The remaining steps were the same as described above for the algal samples.

### DAPI Staining and Fluorescence Microscopy

Fluorescence staining with DAPI was used for nuclear genome size estimation. Briefly, 1 ml of young cultures (2 weeks) of *C. variabilis* NC 64A, SAG 698-1a and SAG 698-1b were each centrifuged and washed with 1× PBS for three times. Supernatant was discarded and the remaining algae were fixed with 4% formaldehyde in 1× PBS for 30 min. The algae were then washed to remove formaldehyde. Then, 0.3% of Triton X-100 was applied to treat algae for 10 min. The cell and nuclear membranes were permeabilized and ready to stain. 500 μl (1 μg/ml DAPI) was used to strain the algae for 10 min in darkness. The fluorescence intensity of stained cells was measured under an Axio Imager 2 microscope (Carl Zeiss Microscopy, LLC) using ImageJ (NIH) software. The reference nuclear genome size of *Chlorella variabilis* NC 64A is 46.2 Mb (Blanc et al., 2010).

### Flow Cytometry

Analysis of SYTOX Green stained nuclei was performed with BD FACSAria II (BD Biosciences, San Jose, United States), a four-laser platform (405, 488, 561, and 642 nm) that is applied for cell sorting and able to detect Forward Scatter (FSC), Side Scatter (SSC), and 17-colors. The SYTOX Green signal was excited by the 488 nm laser, and its emission was detected with a combination of a 505 nm long pass filter and a 530/30 nm band pass filter. A 100 nm nozzle was used with the default sheath pressure of 20 psi, and the sample flow rate was adjusted to a speed of 200–300 nuclei/second. In Total, 10,000 fluorescent particles were measured and recorded. Nuclei of wildtype *A. thaliana* were used as reference. SYTOX Green stained nuclei from the two *Zygnema* strains and *A. thaliana* were first measured separately. Then an appropriate ratio of unstained *A. thaliana* and either SAG 698-1a or SAG 698-1b nuclei was mixed. This ratio was determined based on the individual sample’s event rate with the intent to mix them equally. Once mixed, the nuclei were stained with SYTOX Green and analyzed together. The relative nuclear genome size of green algae was estimated by the known nuclear genome size of *A. thaliana*, 135 Mb^1^. The nuclear genome size of SAG 698-1a or SAG 698-1b was calculated with the formula: nuclear genome size of *Zygnema* (Mb) = (mean G1 peak of *Zygnema* / mean G1 peak of *A. thaliana*) × nuclear genome size of *A. thaliana* (2 × 135 Mb) (Doležel et al., 2007; Pellicer and Leitch, 2014; Wang et al., 2015). *Arabidopsis thaliana* has long been known to exhibit multiple peaks in FC analysis due to endopolyploidy in somatic cells (Galbraith et al., 1991). In the formula, the mean G1 peak of *Arabidopsis thaliana* is the lowest peak from 2C nuclei. *Arabidopsis thaliana* has been used as reference in other studies (Doležel and Bartoš, 2005; Suda et al., 2007; Yoshida et al., 2010, 2020; Bainard and Villarreal, 2013; Galbraith, 2014). It also has the appropriate nuclear genome size to be used as a reference for SAG 698-1a or SAG 698-1b. Three replicates were used for FC analysis.

## Results

As mentioned above, our primary goal is to characterize SAG 698-1a and SAG 698-1b using various experimental approaches. One of the approaches is to determine the nuclear genome size using FC and fluorescence microscopy, which requires an efficient nuclei isolation method (secondary goal). This section is organized to mainly describe the results for the primary goal, while our efforts made for the secondary goal is presented in section “Discussion.”

### SAG 698-1a and SAG 698-1b Have Very Different Cell Size

**FIGURE 1:**
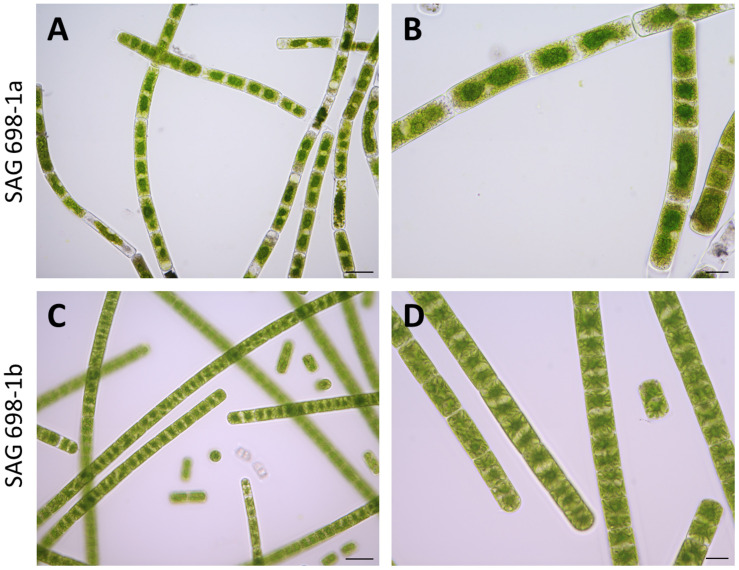
Microscopic images of SAG 698-1a and SAG 698-1b. The algae consist of cylindrical, non-branching filaments cells. Each cell contains two stellate chloroplasts, between which exists one nucleus. Images were taken with an Axio Imager 2 microscope (Carl Zeiss Microscopy, LLC). **(A,B)** SAG 698-1a; **(C,D)** SAG 698-1b. Scale bars: 50 μm **(A,C)** and 20 μm **(B,D)**.

SAG 698-1a and SAG 698-1b were grown using the same standard liquid media, and compared under the microscope for their cell sizes (Table 1). The result showed that the average cell width of SAG 698-1b was 23.16 ± 0.60 μm, very close to Gauch’s (23.4 ± 1.2 μm) (Gauch, 1966) and Miller’s (23.6 ± 0.8 μm) (Miller, 1973) measurements. In contrast, SAG 698-1a exhibited a distinct mean cell width: 26.98 ± 0.97 μm, much larger than SAG 698-1b. In addition, the mean cell length of SAG 698-1a is also ∼2 times of SAG 698-1b (Table 1). The phenotypes of 1 week old cultures of SAG 698-1a and SAG 698-1b are illustrated in Figure 1.

**TABLE 1:** Cell sizes of SAG 698-1a and SAG 698-1b.

	Cell width (μm)		Cell length (μm)	
Cells\Strains	SAG 698-1a	SAG 698-1b	SAG 698-1a	SAG 698-1b
1	27.018	22.621	46.856	48.014
2	25.459	23.237	83.311	40.166
3	26.544	23.872	74.328	47.39
4	26.805	21.783	108.864	33.493
5	26.183	23.048	68.854	26.883
6	26.204	23.066	116.878	43.372
7	25.703	22.547	52.822	33.437
8	26.843	22.364	52.281	34.637
9	27.292	23.089	93.105	37.665
10	28.8	23.305	85.348	30.543
11	28.829	23.922	78.132	32.751
12	26.399	23.573	46.228	34.206
13	27.946	23.38	43.577	39.927
14	27.295	23.791	63.258	41.838
15	27.699	23.872	66.674	31.057
16	26.735	23.169	88.871	33.241
**Mean ± SD**	**26.98 ± 0.97**	**23.16 ± 0.60**	**73.09 ± 22.22**	**36.79 ± 6.12**

### SAG 698-1a Culture Exhibits Morphological Heterogeneity and Cell Size Variation at Different Culture Aage

**FIGURE 2:**
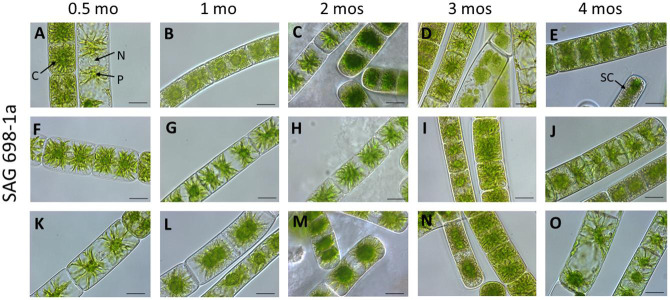
Vegetative cells of SAG 698-1a at different culture ages (time after transfer to fresh medium). **(A,F,K)** 0.5 mo; **(B,G,I)** 1 mo; **(C,H,M)** 2 mos; **(D,L,N)** 3 mos; **(E,J,O)** 4 mos; Arrow pointing labels: C chloroplast; N nucleus; P pyrenoid; and SC storage compounds. Images were taken with a Zeiss Axiovert 200 M (Carl Zeiss, Jena, Germany); scale bars: 20 μm.

**FIGURE 3:**
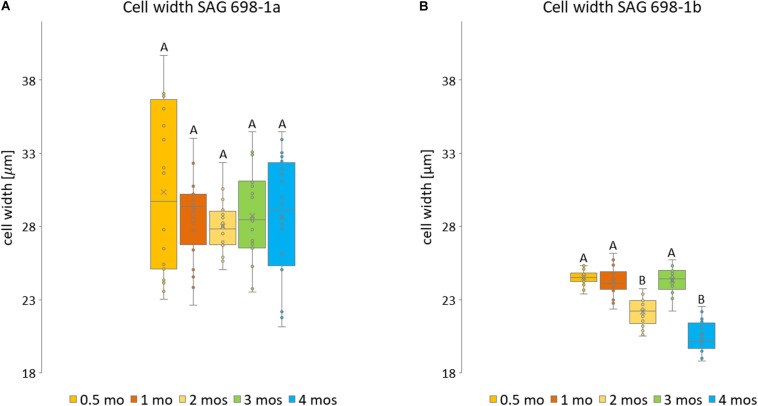
Measurements of SAG 698-1a and SAG 698-1b according to culture age. For each culture age, 20 randomly selected cells from three biological replicates were measured of **(A)** SAG 698-1a and **(B)** SAG 698-1b. Statistically significant differences (multifactor ANOVA, *p* < 0.01) are indicated with different upper-case letters. The detailed info can be found in Supplementary Table 1.

To verify that the SAG 698-1a cultures obtained in 2017 were not contaminated or mis-labeled by ourselves during restocking, we re-obtained SAG 698-1a and SAG 698-1b cultures from SAG in 2019 and viewed the original SAG cultures under the microscope. Interestingly, more heterogeneous cell types were identified for SAG 698-1a (Figures 2, 3A) than for SAG 698-1b (Supplementary Figure 1 and Figure 3B).

Furthermore, we have also studied the cell width variation in relation to the age of the cultured algal filaments (Figure 2; Supplementary Figure 1), and found that the two strains differed significantly (*p* < 0.001) in all ages (Supplementary Table 2). Cells of SAG 698-1a from 0.5-month-old cultures had the greatest average width and standard deviation (30.33 ± 5.8 μm, Figure 3A and Supplementary Table 2). However, the size variation was not significantly different between the culture ages in SAG 698-1a (Figure 3A). In contrast, cells of SAG 698-1b showed significant differences (*p* < 0.001) between the different age stages (Figure 3B). The largest average width in SAG 698-1b was found in 0.5-month-old cultures (24.50 ± 0.52 μm, Figure 3B and Supplementary Table 2).

### DNA Sequence Analyses of Three Marker Genes Suggest That SAG 698-1a Cultures Are Not *Z. circumcarinatum*

As the SAG 698-1a culture displayed a high morphological heterogeneity (Figures 2, 3), we wondered if it was a mixture of different *Zygnema* species rather than a pure culture of *Z. circumcarinatum*. To test this, eight individual filaments were picked with a sterile needle under the dissecting microscope and transferred onto new agar plates. The eight clones were processed to extract genomic DNAs, which were used as the template for PCR amplification of 18S rRNA, *psaA*, and *rbcL* genes and sequenced with Sanger technology. Surprisingly, all the eight clones had the identical sequences of 18S rRNA, *psaA*, and *rbcL* genes. This suggested that these eight purified clones were all identical and the original heterogeneous SAG 698-1a cultures were not a mixture of different species.

More interestingly, the 18S rRNA sequences of the eight clones were identical to two sequences in GenBank: 18S rRNA of *Zygnema cylindricum* SAG 698-2 (accession number: AJ853451.1, 1,758 bp) and 18S rRNA of SAG 698-1a (accession number: KM020155.1, 2,199 bp). Similarly, we found identical hits for our *psaA* and *rbcL* sequences of the eight clones in the plastome of SAG 698-1a (accession number: AY958086.1) published by Turmel et al. (2005). This suggests that our SAG 698-1a is the same culture as previously used for plastome sequencing (Turmel et al., 2005). At the same time, our *psaA* has five mismatches compared to the *psaA* of *Z. cylindricum* SAG 698-2 (EF371262.1, 2,015 bp), and our *rbcL* has two mismatches compared to the *rbcL* of *Z. cylindricum* SAG 698-2 (EF371357.2, 1,353 bp). Therefore, SAG 698-1a is not identical but very similar to SAG 698-2.

Additionally, we also sequenced the three marker genes in our SAG 698-1b culture, and compared them to those in SAG 698-1a and SAG 698-2. The multiple sequence alignments of the three marker genes (Supplementary Figures 2–4) in SAG 698-1a, SAG 698-1b, and SAG 698-2 found many more mismatches between SAG 698-1a and SAG 698-1b than between SAG 698-1a and SAG 698-2. This suggests that our SAG 698-1a culture and the one used in Turmel et al. (2005) are all from a species closely related to *Z. cylindricum* but not *Z. circumcarinatum*. In addition, the *rbcL* gene of UTEX 42 (EF371356.2) has 100% identity with SAG 698-1b *rbcL*, but only 91.25% identity with SAG 698-1a *rbcL*. This is consistent with our recent finding (Orton et al., 2020) that the plastome (accession number: MT040697.1, where *rbcL* is located) of UTEX 1559 (derived from UTEX 42) shares only 85.69% global sequence identity with the that of SAG 698-1a (AY958086.1).

**FIGURE 4:**
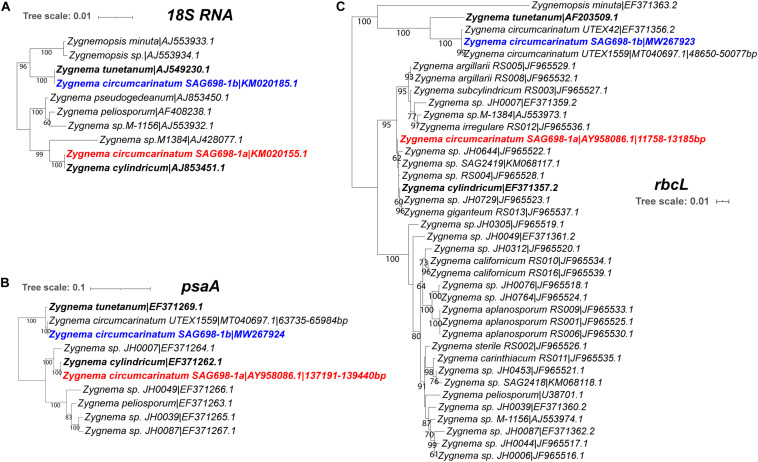
Maximum-likelihood phylogenetic trees of **(A)** 18S rRNA, **(B)** *rbcL*, and **(C)** *psaA* sequences. The detailed method to build these trees are described in Methods. Each sequence has its label including species name and GenBank accession numbers. For SAG 698-1a and UTEX 1559, when the RNA sequences are not available, their plastome accession numbers are provided instead with the positions of the genes indicated. For SAG 698-1b, its *rbcL* (MW267923) and *psaA* (MW267924) mRNA sequences are sequenced in this study and submitted to GenBank.

Furthermore, phylogenetic trees of 18S rRNA, *psaA*, and *rbcL* indicated that SAG 698-1a may be a species very close to *Z. cylindricum* (Figures 4A–C). Similarly, in all the three phylogenies, SAG 698-1b was positioned very close to *Z. tunetanum* (Figures 4A–C), in agreement with the results shown by others and us (Hall et al., 2008; Stancheva et al., 2012; Pichrtová et al., 2018). Notably, *Zygnema tunetanum* was originally described in the genus *Zygogonium* but has been transferred to *Zygnema* based on its phylogenetic position and morphological similarities with *Zygnema* species (Stancheva et al., 2014; Pichrtová et al., 2018). Overall, our strong molecular evidence suggested that SAG 698-1a and SAG 698-1b belong to two distinct clades of *Zygnema* species.

### No conjugation Is Observed Between SAG 698-1a and SAG 698-1b

The significant morphological and molecular differences between SAG 698-1a and SAG 698-1b encouraged us to revisit the conjugation experiments that had been performed by previous studies (Gauch, 1966; Miller, 1973; Miller and Hoshaw, 1974) half a century ago. Using the methods described in these papers, conjugation experiments were conducted with SAG 698-1a and SAG 698-1b cultures. However, after seven days, no conjugation was observed between the two strains (Supplementary Figure 5). One interesting observation was that, under the conjugation conditions, SAG 698-1b filaments fragmentized frequently, while SAG 698-1a did not (Supplementary Figure 5). In our restocking of SAG 698-1b, we noticed that fragmented filaments formed very frequently after transferring from nutrient-rich liquid medium to water agar or to conjugation inducing medium (nutrient-poor). When transferring back into nutrient-rich liquid medium, the fragmented cells turned back to normal filamentous growth again. These data suggest that SAG 698-1b also has very distinct physiological characteristics compared to SAG 698-1a.

### SAG 698-1a Differs in Growth and Photosynthetic Performance and Xanthophyll Cycle Pigments

To further study the physiological properties of the two strains, we have determined the exponential growth by fluorometry and the primary pigment composition, and measured the light-dependent ETRs. The exponential growth phase of both strains lasted approximately 32 days (Supplementary Figure 6) and after this period the cultures were harvested for HPLC analysis. Primary pigment analysis resulted in a similar amount of chlorophyll for both strains (Supplementary Figure 7a and Supplementary Table 3), but differences in xanthophyll cycle pigments, with a higher ratio of zeaxanthin and antheraxanthin to the total xanthophyll cycle pool (A + Z)/(V + A + Z) in strain SAG 698-1a (Supplementary Figure 7b and Supplementary Table 3).

**FIGURE 5:**
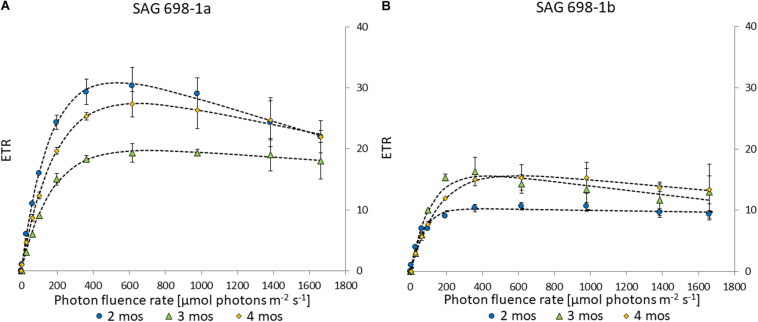
Electron transport rate – irradiance curve of **(A)** SAG 698-1a and **(B)** SAG 698-1b. 2-months old cultures (●); 3-months old cultures (Δ); 4-months old cultures (◆); ETR, electron transport rate.

Electron transport rates can be used to determine the photosynthetic efficiency in *Zygnema* (Herburger et al., 2015). The two strains had similar kinetics and similar progressions across different culture ages (Figure 5). However, strain SAG 698-1a exhibited higher values, with a maximal ETR_max_ of 30.8 ± 2.76 in 2 months old cultures (Figure 5A and Supplementary Table 4), while SAG 698-1b reached the highest ETR_max_ in 3 months old cultures with only 16.3 ± 1.80 (Figure 5B and Supplementary Table 4). ETR_max_ values measured at different time points (2–4 months) of SAG 698-1b showed little variation between the culture ages (Figure 5B and Supplementary Table 4). All ETR_max_ values differed significantly (*p* < 0.05) between the two strains, when the individual culture ages were compared.

### SAG 698-1a Differs in Mucilage Sheath and Chloroplast Shapes From SAG 698-1b

**FIGURE 6:**
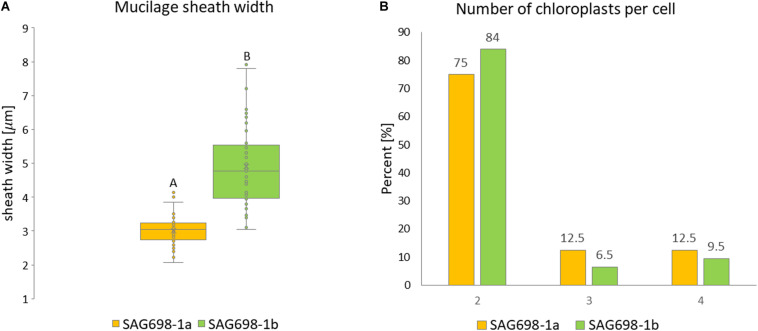
**(A)** Mucilage sheath width. Statistically significant differences (Mann–Whitney-*U*-test, *p* < 0.001) are indicated by different upper-case letters. **(B)** Chloroplast number per cell.

The mucilage sheath width was significantly thicker in filaments of SAG 698-1b (Figure 6A, Supplementary Figure 8, and Supplementary Table 2). The mean mucilage sheath of this strain was 4.90 ± 1.17 μm while SAG 698-1a had a mean mucilage sheath width of 3.01 ± 0.43 μm (Figure 6A and Supplementary Table 2). When viewed by a Zeiss Filter Set 01 (Ex: BP 365/12 nm, Em: LP 397 nm), the calcofluor white staining showed blue fluorescence of cell walls indicating cellulose and callose, and the red fluorescence indicated chlorophyll autofluorescence (Supplementary Figure 8). The two strains had a similar number of chloroplasts per cell: in 75 or 84% (strain SAG 698-1a and SAG 698-1b, respectively) of the cells, two chloroplasts per cell were found, but also three and four chloroplasts per cell could be observed in a lower percentage of cells (Figure 6B). Moreover, confocal laser scanning images of the chloroplast autofluorescence allowed to depict a clearly different chloroplast shape between the two strains (Supplementary Figure 9).

### SAG 698-1a and SAG 698-1b Are Estimated to Have Very Different Nuclear Genome Sizes

**FIGURE 7:**
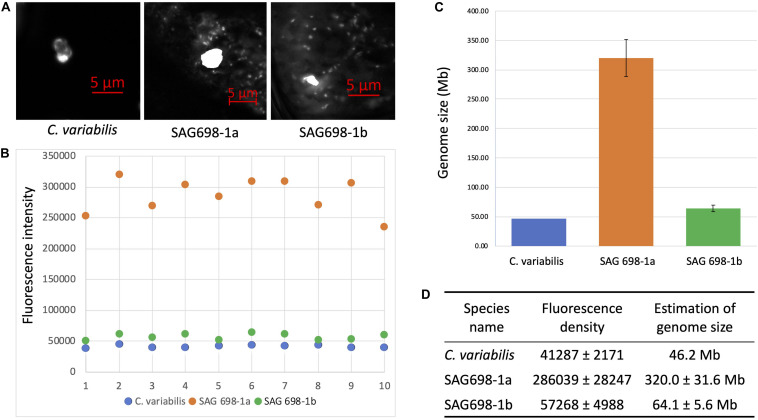
DAPI staining DNA fluorescence analysis of SAG 698-1a and SAG 698-1b. **(A)** DAPI stained nucleus images of *Chlorella variabilis*, SAG 698-1a and SAG 698-1b; **(B)** Fluorescence intensity of nuclei in the cells; **(C,D)** Genome size calculation of SAG 698-1a and SAG 698-1b (mean values ± SD) using *C. variabilis* as the reference (known genome size).

The nuclear genome sizes of SAG 698-1a and SAG 698-1b were first estimated by a microspectrophotometry method. Using DAPI staining, the density signals showed that SAG 698-1a and SAG 698-1b had very different DNA content and thus different nuclear genome sizes, estimated at 320.0 ± 31.6Mb (coefficient of variation = 0.10) and 64.1 ± 5.6 Mb (coefficient of variation = 0.09), respectively (Figure 7).

**FIGURE 8:**
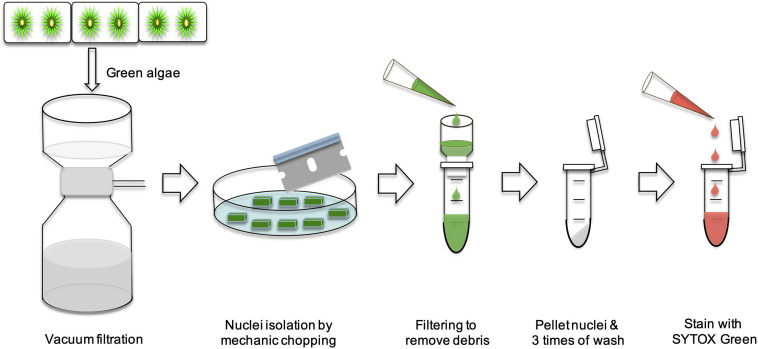
Schematic diagram of Flow cytometry analysis in SAG 698-1a and SAG 698-1b.

**FIGURE 9:**
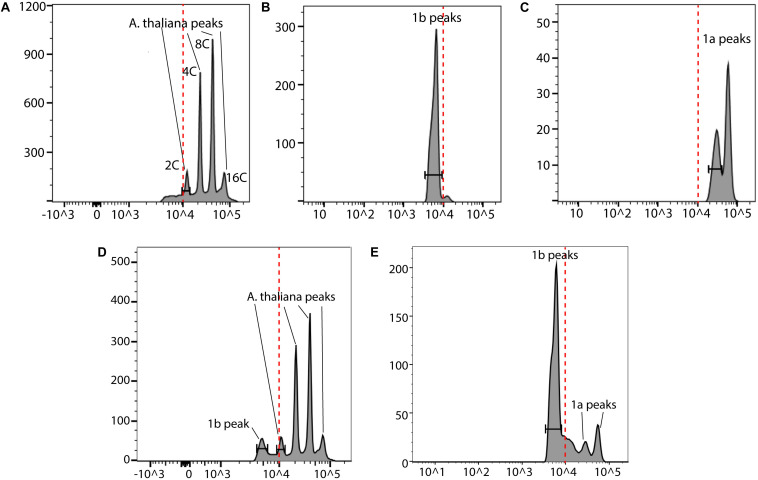
Flow cytometry analysis of SAG 698-1a and SAG 698-1b. **(A)** Cell cycle G1 phase nuclei of *A. thaliana*; **(B)** Cell cycle G1 phase nuclei of SAG 698-1b; **(C)** Cell cycle G1 phase nuclei of SAG 698-1a; **(D)** Cell cycle G1 phase mixed nuclei of *A. thaliana* and SAG 698-1b; **(E)** Cell cycle G1 phase mixed nuclei of SAG 698-1a and SAG 698-1b. *X*-axis is the fluorescence intensity representing the relative nuclear DNA content, and *Y*-axis is the channel bins representing the count of nuclei. Red dash lines indicate the fluorescence intensity at 10^4^.

In order to further validate our DAPI results, a fast FC method was developed (Figure 8). We used *A*. *thaliana* as the reference in our FC analyses. The nuclei from these three species were first analyzed separately (Figures 9A–C). Then an appropriate ratio of nuclei from two species were mixed (Figure 9D for *A. thaliana* and SAG 698-1b; Figure 9E for SAG 698-1b and SAG 698-1a). Note that although SAG 698-1b had one large peak and one very small peak (Figure 9B) in terms of DNA content, *A. thaliana* had four peaks (Figure 9A) and SAG 698-1a had two peaks (Figure 9C). The FC results further suggested that SAG 698-1a and SAG 698-1b have very different nuclear genome sizes of 313.2 ± 2.0 Mb (coefficient of variation = 0.006) and 63.5 ± 0.5 Mb (coefficient of variation = 0.008), respectively, which are very close to the above DAPI estimations. The large difference between nuclear genome size of SAG 698-1a and SAG 698-1b strengthened the fact that SAG 698-1a is not the same species as SAG 698-1b.

## Discussion

Sequencing the genomes of *Z. circumcarinatum* can provide new insights into the early evolution of land plants. To assist the genome sequencing project, in the present study, we have collected numerous morphological, morphometrical, physiological and molecular data to characterize two *Zygnema* strains SAG 698-1a and SAG 698-1b. Particularly, SAG 698-1a had previously been used for the analysis of chloroplast genomes (Turmel et al., 2005) and of transcriptomes (de Vries et al., 2018). As there were doubts concerning the species assignment of SAG 698-1a (Stancheva et al., 2012; Orton et al., 2020), this study was needed to better characterize this strain. Historically, different strains of *Z. circumcarinatum* were isolated by Viktor Czurda (1930) who described them with a cell width of 20–22 μm, and performed numerous conjugation experiments. He isolated 10 clones (a–j) and combined these in three independent experimental setups with 57–104 individual experiments. With these experiments it became clear that clone “a” was (+) and clone “b” was (−) mating type (Czurda, 1930). Then, (Gauch, 1966) and (Miller, 1973) had observed conjugations between SAG 698-1a and SAG 698-1b regardless of their past history. Notably, according to https://sagdb.uni-goettingen.de/detailedList.php?str_number=698-2, SAG 698-2 (*Z. cylindricum*) was also isolated by Viktor Czurda. SAG 698-2 should be one of the 10 clones that were experimented for conjugation by Czurda, but not the clone “a” or clone “b”.

### Sequence Analysis Provides Strong Evidence That SAG 698-1a and SAG 698-1b Are Two Different Species

We observed different morphological phenotypes of filaments in the SAG 698-1a cultures (Figure 2). Further DNA sequencing and comparison of 18S rRNA, *rbcL* and *psaA* genes in SAG 698-1a and SAG 698-1b revealed that they have 41, 118, and 206 base pairs difference, respectively, between the full-length genes of the two strains. Among the three marker genes, *psaA* gene is more variable than *rbcL* and then 18S rRNA, so it should be more useful for distinguishing closely related species within *Zygnema*. However, species delimitation requires more rigorous analyses than the marker sequence comparison alone. The reason is that no appropriate cutoff exists to define two species, i.e., how many base pair differences in which marker genes would be sufficient to define two different species.

Furthermore, in the 18S rRNA phylogeny as well as the *psaA* and *rbcL* phylogenies, SAG 698-1a was always positioned closely with *Z. cylindricum* in the same clade (Figure 4). In contrast, SAG 698-1b formed another clade together with *Z. tunetanum* (Figure 4). Previous phylogenetic studies using different marker genes (*psaA, rbcL*, and *cox*III) have shown that there might be at least two different clades within the genus *Zygnema* (Hall et al., 2008; Stancheva et al., 2012; Pichrtová et al., 2018), one of which contains SAG 698-1a. Pichrtová et al. even indicated that there are three *Zygnema* clades based on a *rbcL* phylogeny (Pichrtová et al., 2018), which agrees with our Figure 4C. Overall, our phylogenetic analysis with the three marker genes confirmed the findings of previous studies that SAG 698-1a may be a species closely related to *Z. cylindricum*, and SAG 698-1b may be a species closely related to *Z. tunetanum*. Therefore, these two SAG strains clearly belong to two different clades of the genus *Zygnema* (Figure 4).

### Cell Morphology of SAG 698-1a and 698-1b Are Markedly Different

Although it is now very clear that SAG 698-1a and SAG 698-1b are different species, in this study we do not intend to taxonomically reclassify (or name) the two strains. However, we hope to address the question: which of the two strains should keep the species name: *Z. circumcarinatum*? Apparently, *Z. circumcarinatum* has been extensively studied by Gauch and Miller (Gauch, 1966; Miller, 1973; Miller and Hoshaw, 1974) half a century ago, and in their studies the descriptions of *Z. circumcarinatum* clearly match more our SAG 698-1b than our SAG 698-1a. The reason is that in our morphological observation, SAG 698-1b had a typical cell width of *Z. circumcarinatum* that was reported by Gauch and Miller (Gauch, 1966; Miller and Hoshaw, 1974) and initially described by Czurda (1930). Unexpectedly, our SAG 698-1a was on average ∼4 μm larger than the values reported by Gauch and Miller, with a high variation in width. This suggests that the SAG 698-1a cultures we obtained from SAG independently in 2017 and 2019 are different from the strain investigated by Gauch (1966) and Miller (1973). In addition, the cell length of SAG 698-1a investigated in the present study is roughly two times that of SAG 698-1b (Table 1). Therefore, we believe that it is more appropriate to keep naming SAG 698-1b *Z. circumcarinatum*, while SAG 698-1a should be renamed as a different species. Meanwhile, as *Z. tunetanum* and SAG 698-1b are very close in sequence and in phylogeny for all three marker genes, this also echoes recent studies (Stancheva et al., 2014; Pichrtová et al., 2018), which renamed *Zygogonium tunetanum* as *Z. tunetanum*.

Additionally, the cell size differences of SAG 698-1a and SAG 698-1b, together with the observation that these strains could not conjugate, indicate that changes might have happened in maintaining the SAG 698-1a cultures at the algal collection centers in the past half century. The fact that conjugation could not be induced in the present study, also prevented a classical morphological determination of *Z. circumcarinatum* based on the zygospore architecture (Stancheva et al., 2012). Actually, the lack of zygospores prevents a formal reclassification and determination on which of the two strains is *Z. circumcarinatum sensu* Czurda(Czurda, 1930).

### Differences Exist in Physiological Performance Between the Strains

The two strains were reported to have different pyrenoid structures (Gauch, 1966), and SAG 698-1a was greener than SAG 698-1b (Miller, 1973) in appearance when investigating the chlorophyll contents by thin layer chromatography. In order to reinvestigate this description, we performed a primary pigment analysis, and found a similar amount of chlorophyll a and b for both strains (Supplementary Figure 7a and Supplementary Table 3). However, we observed differences in the xanthophyll cycle pigments, with a higher ratio of zeaxanthin and antheraxanthin in SAG 698-1a (Supplementary Figure 7b and Supplementary Table 3), leading to a higher DEPS. In fact, the DEPS found in SAG 698-1a is similar with what has been reported for UV-treated *Zygnema* strains (Pichrtová et al., 2013), whereas SAG 698-1b reassembled more the control condition of *Zygnema* analyzed by Pichrtová et al. (2013) and Holzinger et al. (2018). These observations go along with the finding that SAG 698-1a always had higher values in ETR_max_ under the same cultivation condition (Figure 5), suggesting a clear physiological difference between the two strains. SAG 698-1a has recently been used for transcriptomic analysis (de Vries et al., 2018), who also performed a physiological characterization of this strain, showing that at ∼600 μmol photons m^–2^ s^–1^ the ETR was saturated. Earlier investigations of different *Zygnema spp*. showed species-specific differences of ETR_max_ values (Herburger et al., 2015). Therefore, this value can be used to describe physiological differences between strains cultivated under the same conditions.

### Nuclear Genome Size Evaluation Shows Marked Differences Between the Two Strains

As our secondary goal in this study, we also reported an easy and fast method to estimate the nuclear genome sizes of SAG 698-1a and SAG 698-1b, which can also be applied to other filamentous Zygnematophyceae. With two different methods (DAPI staining in Figure 7, and FC in Figure 9), we observed that the genome sizes of the two strains must be very different: 313.2 ± 2.0 Mb for SAG 698-1a and 63.5 ± 0.5 Mb for SAG 698-1b, according to the more accurate FC estimation.

In order to obtain enough high-quality nuclei for FC and DAPI analysis, we have also tried generating protoplasts following the methods described by Mazalová et al. (2011) and Ohiwa (1977, 1981). Unfortunately, these methods failed as the cell walls of SAG 698-1b were particularly hard to be removed with cell wall digesting enzymes (cellulases, hemicellulases and pectinases) (Supplementary Figure 10). The thicker mucilage layer in SAG 698-1b (Figure 6A) may help to partially explain difficulties in DNA extraction compared to SAG 698-1a. This is most likely caused by the high abundance of homogalacturonan and arabinogalactan proteins (AGPs) in the mucilage layer of Zygnematopyhceae (Holzinger and Pichrtová, 2016; Herburger et al., 2019; Palacio-López et al., 2019). Indeed, in the transition from water to land, the highly complex cell walls of early land plants (Zygnematophyceae like) may have played essential roles for the adaptation to various environmental stresses, such as UV, water loss, and cold (Sørensen et al., 2010, 2011).

Previous reports have shown that the DNA content of *Z. circumcarinatum* CAUP K402a (supposedly identical to SAG 698-1a) was 3.07 ± 0.06 pg (Mazalová et al., 2011), which would account for a much higher nuclear genome size in the range of 3,000 Mb. Kapraun DF also showed that the 2C DNA content of *Zygnematales* ranged from 0.5 to 4.2 pg (Kapraun, 2005). It is unclear why CAUP K402a, SAG 698-1a, and SAG 698-1b have very different nuclear genome sizes if they were all derived from the original isolates by Czurda (1930). However, it is possible that they were actually of different *Zygnema* species as presented in this paper. The other possibility is due to the previously used protoplast generation method for FC analysis (Mazalová et al., 2011). Various studies have shown that protoplasts had drawbacks in the FC analysis, such as unpredicted position of nuclei, nonspecific binding, interferences from pigments, organelles and secondary metabolites (Loureiro et al., 2006; Doležel et al., 2007; Bennett et al., 2008; Cires et al., 2011; Wang et al., 2015). From our DAPI staining results (Figure 7), DNAs from chloroplast and mitochondria (numerous smaller staining spots around the nucleus) were clearly observed, indicating that interference from organellar fluorescence are very likely. Our nuclear genome size estimations for SAG 698-1a and SAG 698-1b with three FC replicates (Figure 9) produced very consistent results, with much smaller standard deviations (313.2 ± 2.0 Mb for SAG 698-1a and 63.5 ± 0.5 Mb for SAG 698-1b) than DAPI estimations. The larger standard deviations (320.0 ± 31.6 and 64.1 ± 5.6 Mb) from DAPI estimations may be caused by the staining dye, as (Doležel and Bartoš, 2005) indicated DAPI preferentially binds to AT-rich DNA regions, which may lead to erroneous results.

In our simple high-quality nuclei extraction protocol, some steps are important for obtaining desired results. First, young and fresh algae are preferred, because old algal cells produce more background signals (Pellicer and Leitch, 2014). For the nucleus isolation buffer, we used the buffer from the commercial kit, but it is recommended to also try other nuclei extraction buffers to find the most suitable ones (Doležel and Bartoš, 2005; Pellicer and Leitch, 2014). In our study, we centrifuged and washed the nuclei pellet for three times in order to remove the debris, organelles and cytosolic compounds. Particularly, secondary metabolites and mucilaginous materials of algae can interfere and reduce the accuracy of FC results (Loureiro et al., 2006; Bennett et al., 2008; Cires et al., 2011; Wang et al., 2015). The 3 times washing step is critical for removing background signals and improving the accuracy of nuclear genome size estimation. The quality of extracted nuclei is very important for FC analysis, but appropriate fluorescent staining also has a significant impact (Doležel and Bartoš, 2005). Various fluorescent dyes, such as EtBr, DAPI, PI, and SYTOX Green, have been used in DNA staining. Furthermore, appropriate staining time, and the ratio of fluorescent dye and nuclei should also be taken into consideration. For example, we stained the nuclei with PI for 1h following the protocol of the kit, but found that the nuclei suspension produced more noisy signals from the debris (Doležel et al., 2007; Wang et al., 2015). In our study, 2∼5 min of PI or SYTOX Green staining produced better results.

## Conclusion

In this study, we provided multiple lines of evidence to show that SAG 698-1a is most likely a *Z. cylindricum* related species. However, we cannot resolve when this error occurred, but at least the data provided by Turmel et al. (2005) suggest that it had happened before 2005. We showed a new method for determining nuclear genome sizes, which differed largely between these two strains, being 313.2 ± 2.0 Mb for SAG 698-1a and 63.5 ± 0.5 Mb for SAG 698-1b. The present study provides a good basis for the *Zygnema* draft genomes that we and others are currently developing.

## Data Availability Statement

The original contributions presented in the study are included in the article/Supplementary Material, further inquiries can be directed to the corresponding author/s.

## Author Contributions

XF, YY, and AH designed the study. XF performed the experiments (molecular biology, morphometry, and conjugation test). DA performed molecular analysis. CP performed physiology and morphometry. XF, DA, AH, and YY wrote the manuscript. All authors read and approved the final manuscript.

## Conflict of Interest

The authors declare that the research was conducted in the absence of any commercial or financial relationships that could be construed as a potential conflict of interest.

## Footnotes

1 https://www.arabidopsis.org/portals/genAnnotation/gene_structural_annotation/agicomplete.jsp

## Supplementary Material

Supplementary Figure 1 Vegetative cells of SAG698-1b at different culture ages (time after transfer to fresh medium). **(a,f,k)** 0.5 mo; **(b,j,l)** 1 mo; **(c,h,m)** 2 mos; **(d,i,n)** 3 mos; **(e,j,o)** 4 mos; Arrow pointing labels: C chloroplast; N nucleus; P pyrenoid; and SC storage compounds. Images were taken with a Zeiss Axiovert 200M (Carl Zeiss, Jena, Germany); scale bars: 20 μm.

Supplementary Figure 2 Alignment of partial 18S rRNA from SAG 698-1a, SAG 698-1b, and SAG 698-2.

Supplementary Figure 3 Alignment of partial *psaA* from SAG 698-1a, SAG 698-1b, and SAG 698-2.

Supplementary Figure 4 Alignment of partial *rbcL* from SAG 698-1a, SAG 698-1b, and SAG 698-2.

Supplementary Figure 5 Conjugation test of SAG 698-1a and SAG 698-1b. **(a)** Mixture of SAG 698-1a and SAG 698-1b in C-medium for 3 days; **(b)** Mixture in C-medium for 7 days; FM: fragmentation. The long filaments are all from SAG 698-1a, while the short and fragmented filaments are from SAG 698-1b. However, no conjugations between the two strains were observed. Images were taken with an Axio Imager 2 microscope (Carl Zeiss Microscopy, LLC). Scale bars: 50 μm.

Supplementary Figure 6 Exponential growth of *Zygnema.* **(a)** SAG 698-1a; **(b)** SAG 698-1b.

Supplementary Figure 7 **(a)** Chlorophyll amount and **(b)** xanthophyll cycle pigments of strains SAG 698-1a and SAG 698-1b. Statistical evaluation of the chlorophyll amounts was performed with Mann–Whitney-*U*-test; no significant difference (*p* > 0.05) was found for chl a and chl b.

Supplementary Figure 8 Stained filaments of *Zygnema*. **(a–d)** Indian ink stained cells; **(e–h)** Calcofluor white stained cells. Photos were taken with Zeiss Filter set 1, excitation: band pass (BP) 365/12 nm and emission: long pass (LP) 397 nm. **(a,b,e,f)** SAG 698-1a; **(c,d,g,h)** SAG 698-1b; Images were taken with a Zeiss Axiovert 200M (Carl Zeiss, Jena, Germany); scale bars: 20 μm.

Supplementary Figure 9 Confocal laser scanning images of *Zygnema*; **(a,b)** SAG 698-1a; **(c,d)** SAG 698-1b; Images were taken with a Zeiss Axiovert 200M, Pascal LSM5 (Carl Zeiss, Jena, Germany); scale bars: 20 μm.

Supplementary Figure 10 Microscope image of SAG 698-1a and SAG 698-1b filaments plasmolyzed and digested with cell wall enzymes. Images were taken under Axio Imager 2 microscope. **(a,b)** SAG 698-1a; **(c,d)** SAG 698-1b. Scale bar in **(a)**: 50 μm, in **(b–d)**: 20 μm.

Supplementary Table 1 PCR primers used for sequencing.

Supplementary Table 2 Measurements of cell diameter and mucilage sheath width, Q = quartile; IQR = inter quartile range; SD = standard deviation, SE = standard error.

Supplementary Table 3 HPLC pigment analysis of SAG698-1a and SAG698-1b, Statistical level of significance of the pigment amounts (Mann–Whitney-*U*-test); ^∗^*p* < 0.05; ^∗∗^*p* < 0.01; ^∗∗∗^*p* < 0.001.

Supplementary Table 4 PAM (pulse-amplitude modulated fluorimeter) measurements of *Zygnema* SAG 698-1a and SAG 698-1b with increasing photon fluence rates up to 1661 μmol photons m^–2^ s^–1^; α: curve slope of the initial linear light-limited part; lc: light compensation point; ETRmax: maximum electron transport rate.
