# Supplementary figures and images for: Characterization of Two Zygnema Strains (Zygnema circumcarinatum SAG 698-1a and SAG 698-1b) and a Rapid Method to Estimate Nuclear Genome Size of Zygnematophycean Green Algae
Source: Front Plant Sci. 2021 Feb 10;12:610381. doi: 10.3389/fpls.2021.610381 (PMC7902510; doi:10.3389/fpls.2021.610381)

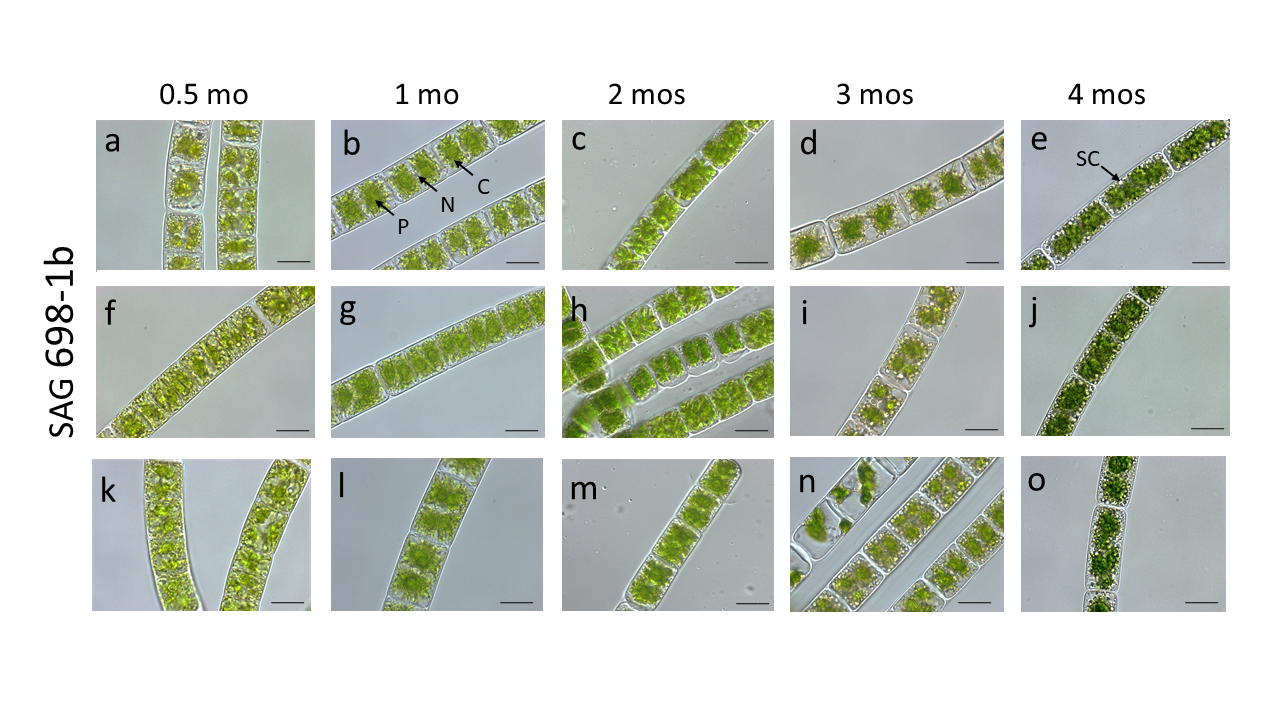

Supplement: Supplementary Figure 1 — Vegetative cells of SAG698-1b at different culture ages (time after transfer to fresh medium). (a,f,k) 0.5 mo; (b,j,l) 1 mo; (c,h,m) 2 mos; (d,i,n) 3 mos; (e,j,o) 4 mos; Arrow pointing labels: C chloroplast; N nucleus; P pyrenoid; and SC storage compounds. Images were taken with a Zeiss Axiovert 200M (Carl Zeiss, Jena, Germany); scale bars: 20 μm. [file Image_1.TIF]

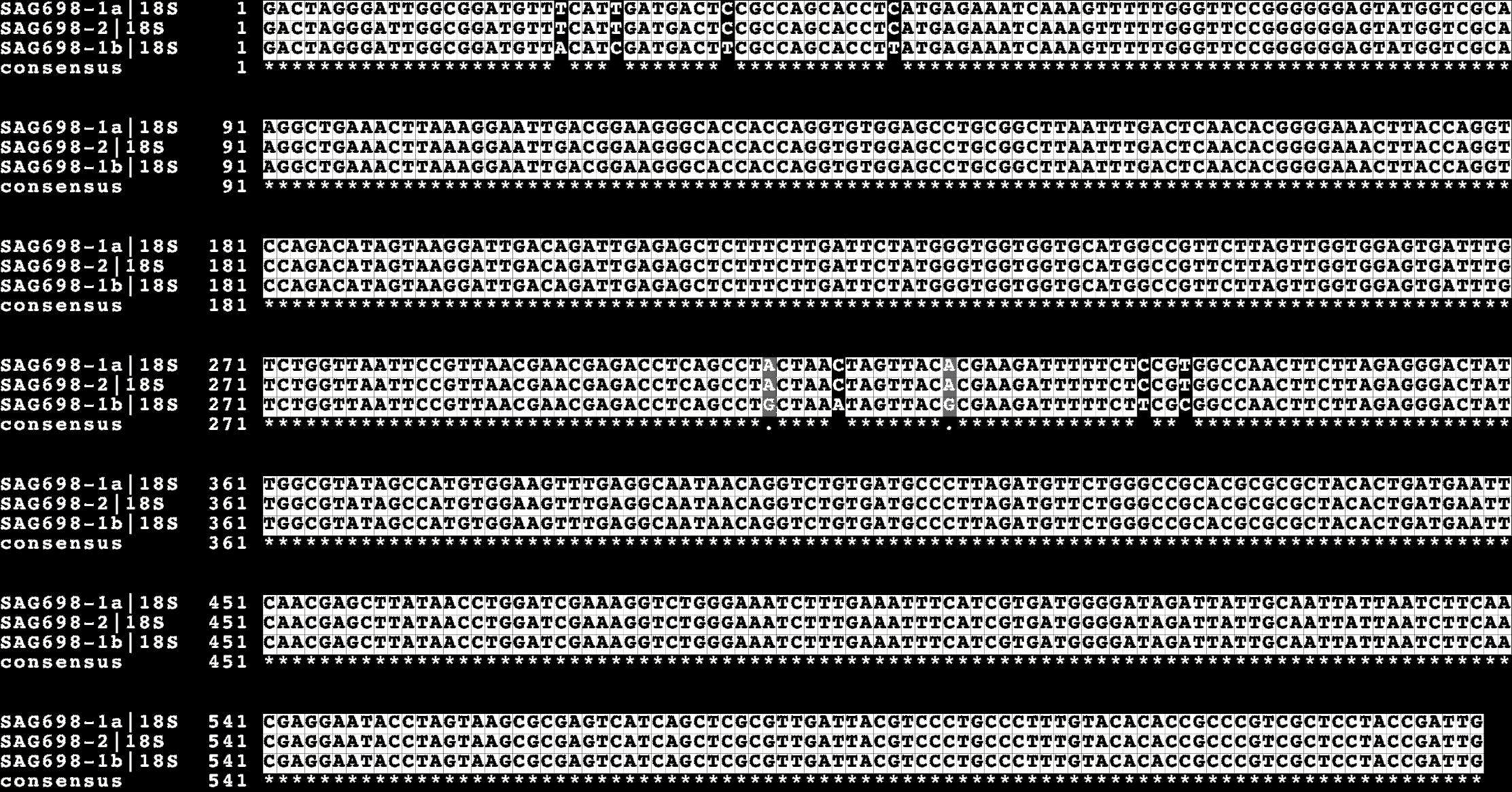

Supplement: Supplementary Figure 2 — Alignment of partial 18S rRNA from SAG 698-1a, SAG 698-1b, and SAG 698-2. [file Image_2.TIF]

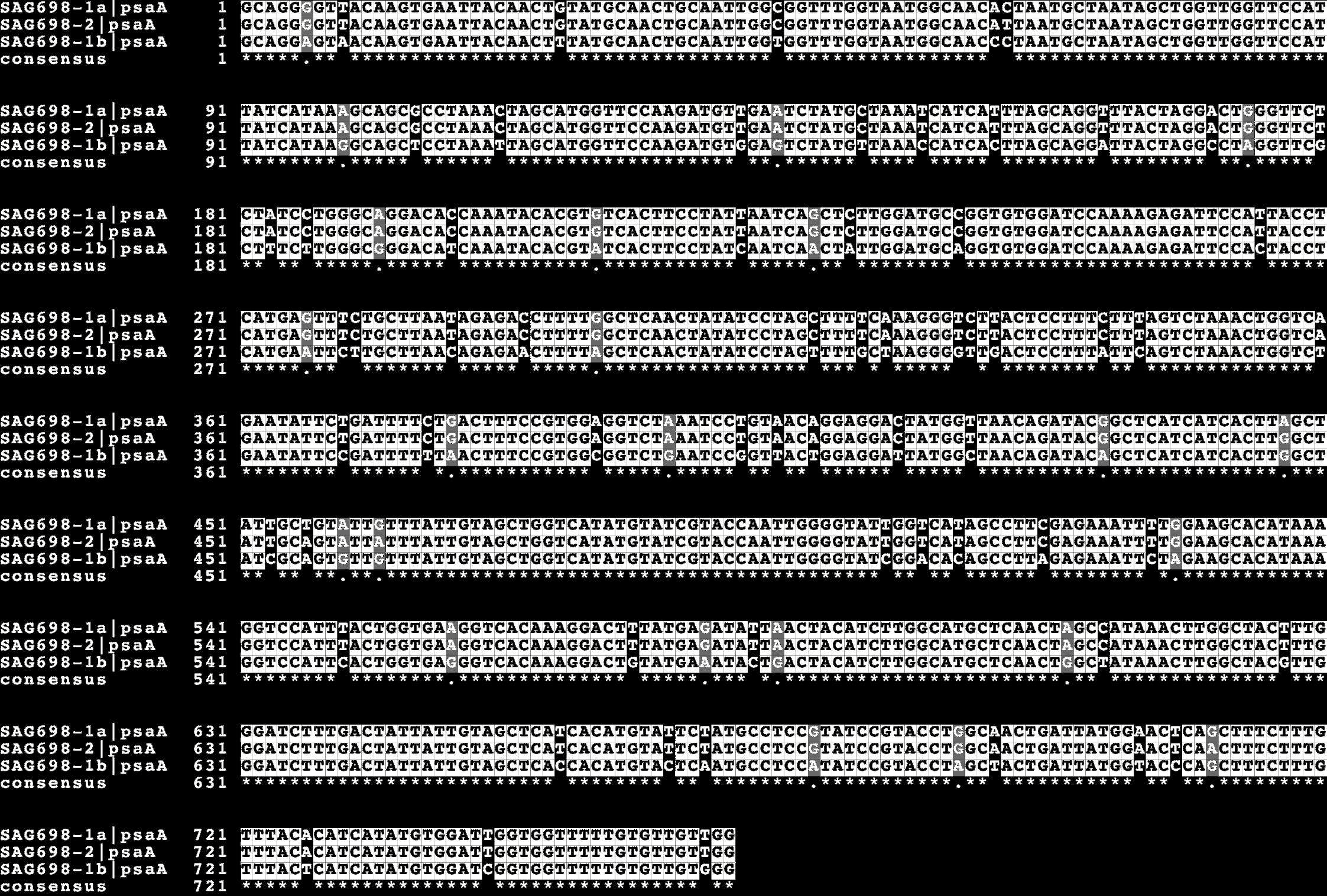

Supplement: Supplementary Figure 3 — Alignment of partial psaA from SAG 698-1a, SAG 698-1b, and SAG 698-2. [file Image_3.TIFF]

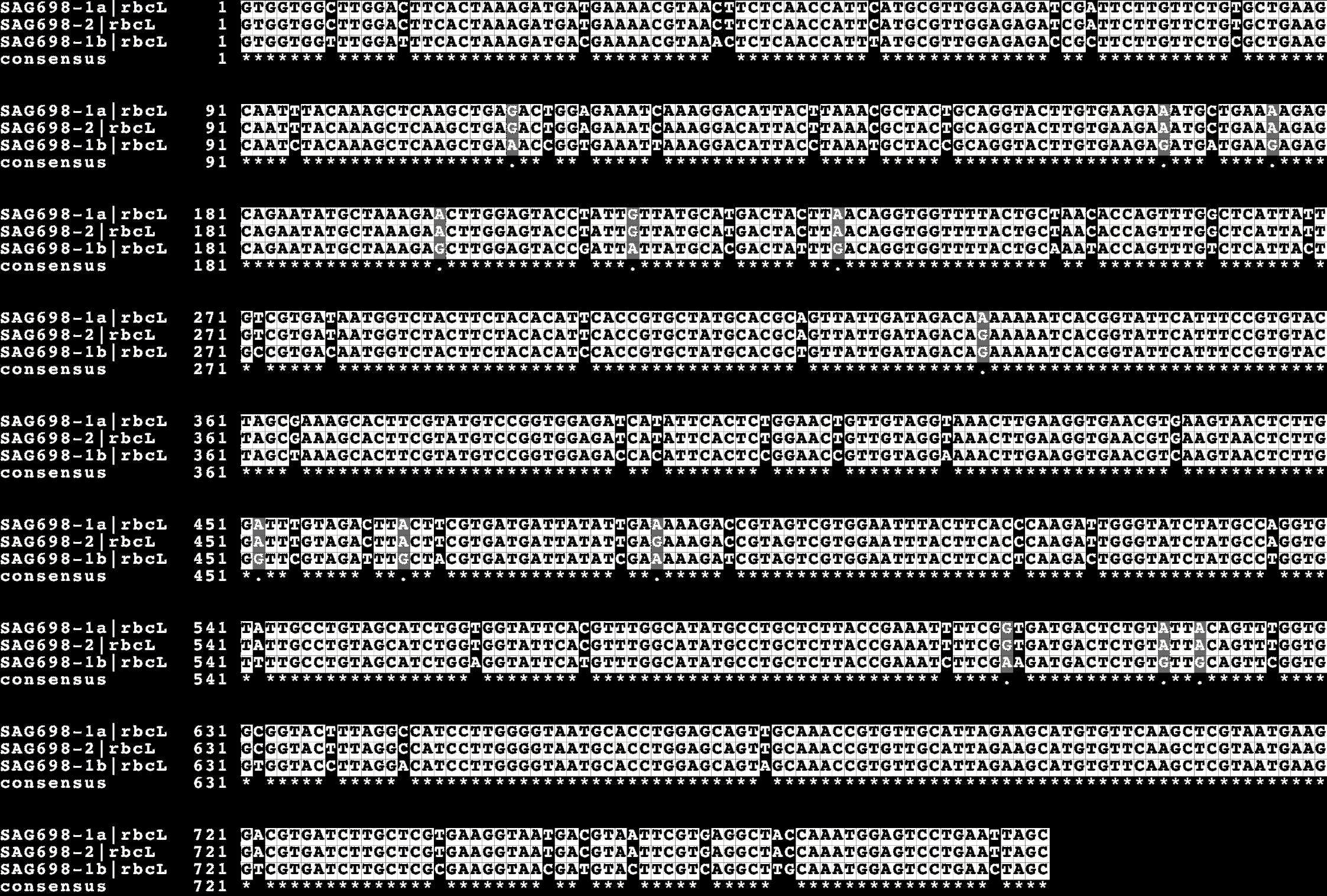

Supplement: Supplementary Figure 4 — Alignment of partial rbcL from SAG 698-1a, SAG 698-1b, and SAG 698-2. [file Image_4.TIF]

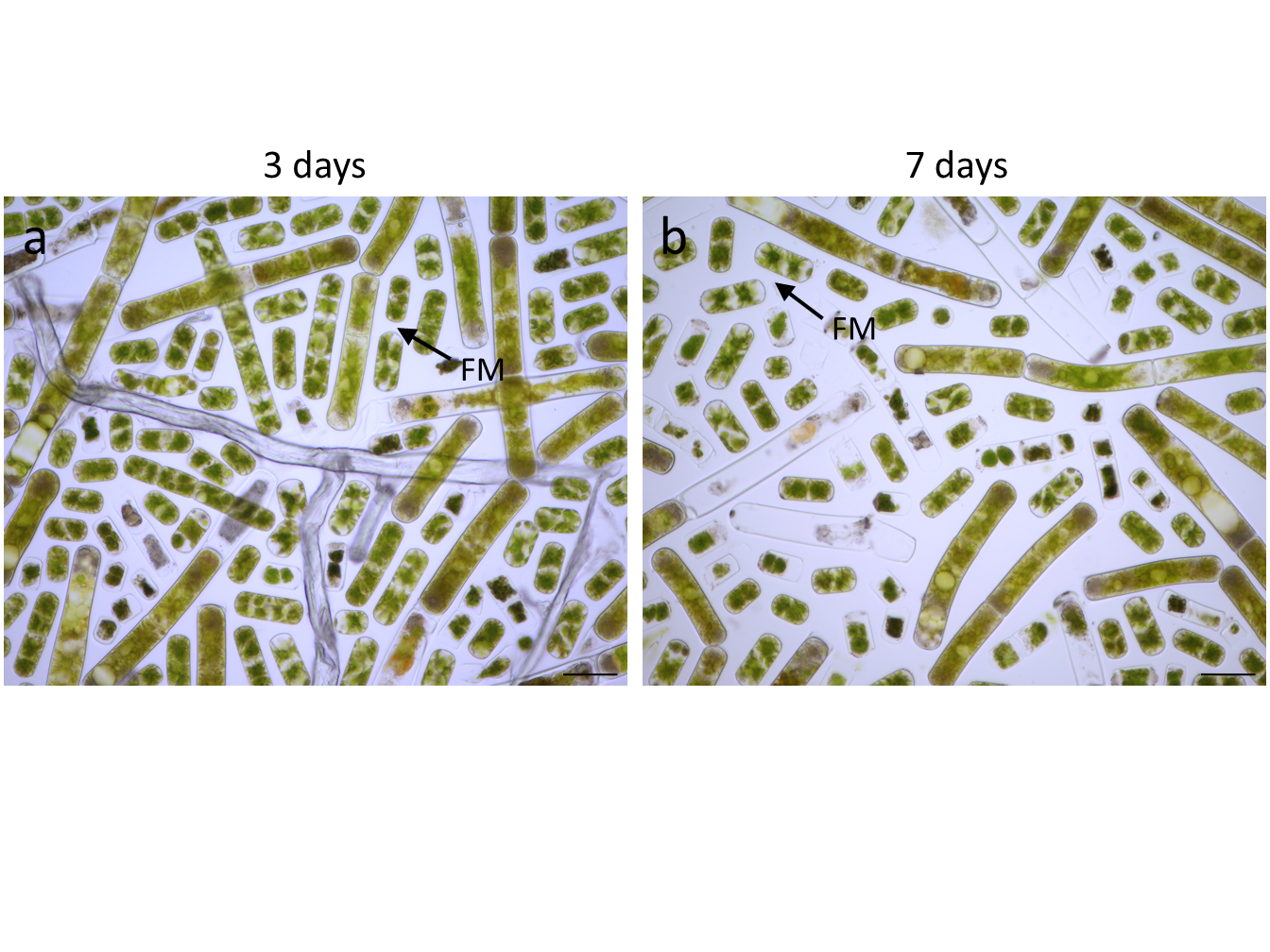

Supplement: Supplementary Figure 5 — Conjugation test of SAG 698-1a and SAG 698-1b. (a) Mixture of SAG 698-1a and SAG 698-1b in C-medium for 3 days; (b) Mixture in C-medium for 7 days; FM: fragmentation. The long filaments are all from SAG 698-1a, while the short and fragmented filaments are from SAG 698-1b. However, no conjugations between the two strains were observed. Images were taken with an Axio Imager 2 microscope (Carl Zeiss Microscopy, LLC). Scale bars: 50 μm. [file Image_5.TIF]

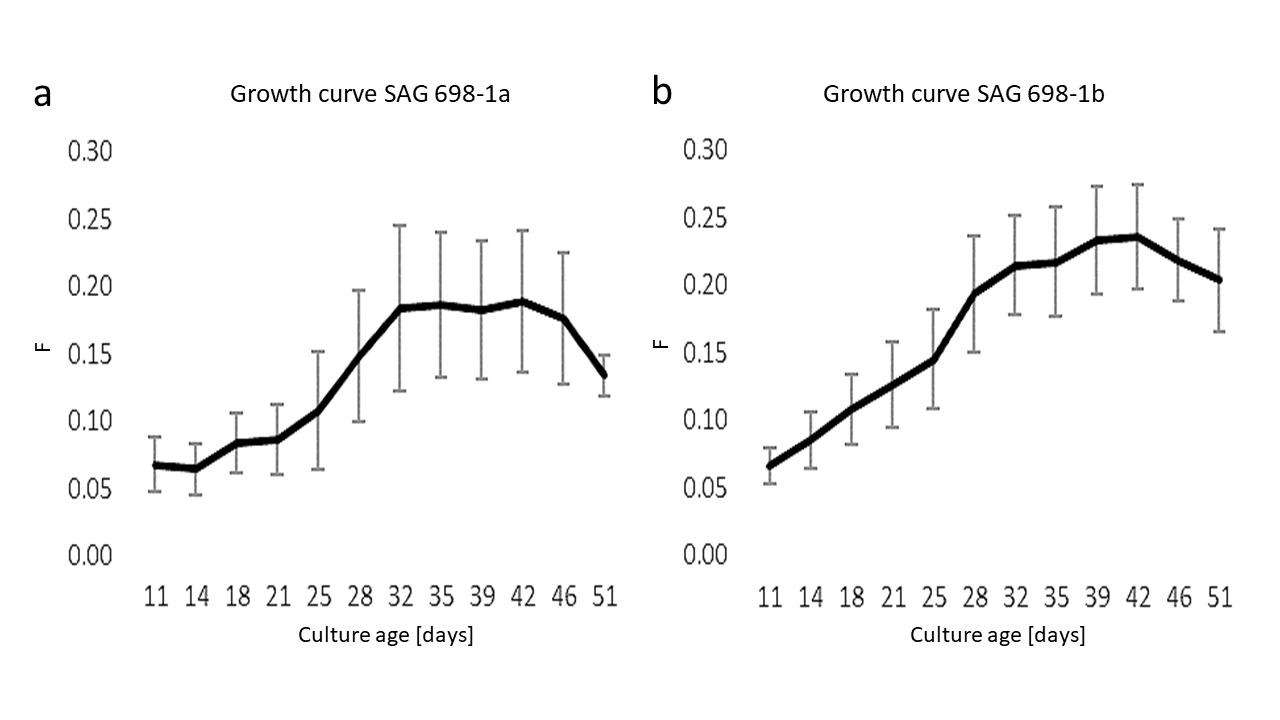

Supplement: Supplementary Figure 6 — Exponential growth of Zygnema. (a) SAG 698-1a; (b) SAG 698-1b. [file Image_6.TIF]

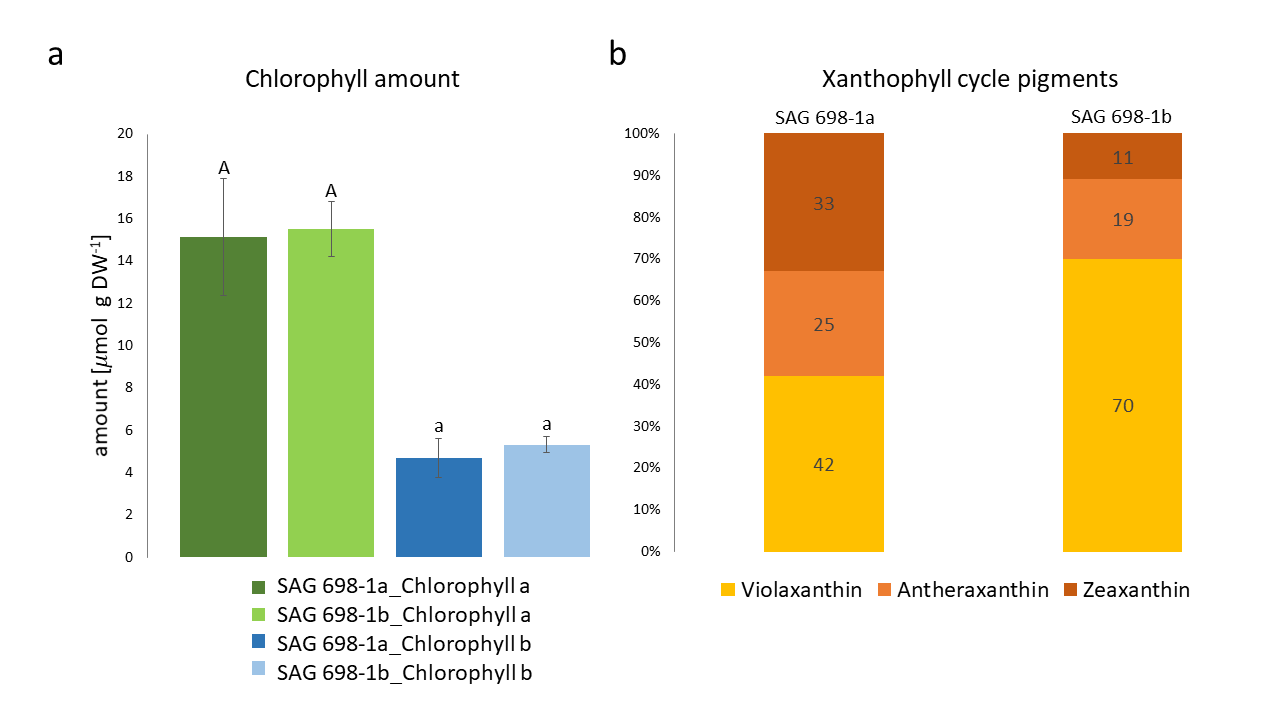

Supplement: Supplementary Figure 7 — (a) Chlorophyll amount and (b) xanthophyll cycle pigments of strains SAG 698-1a and SAG 698-1b. Statistical evaluation of the chlorophyll amounts was performed with Mann–Whitney-U-test; no significant difference (p > 0.05) was found for chl a and chl b. [file Image_7.tif]

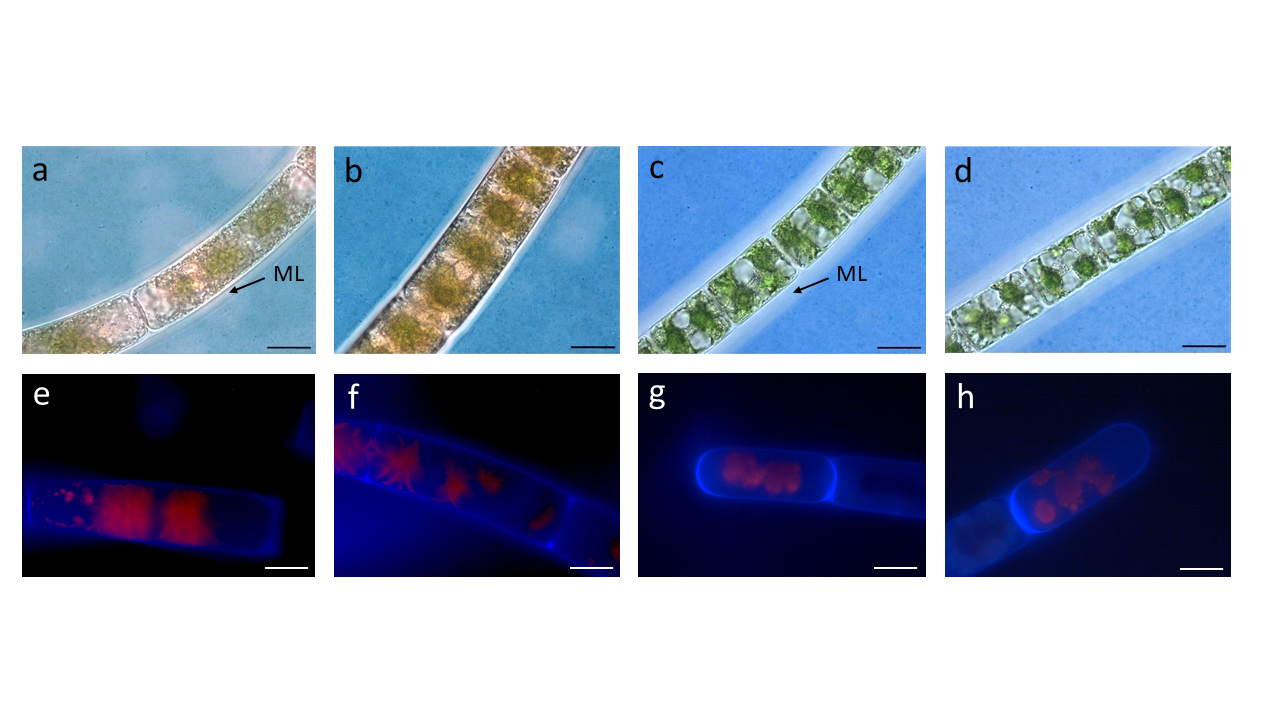

Supplement: Supplementary Figure 8 — Stained filaments of Zygnema. (a–d) Indian ink stained cells; (e–h) Calcofluor white stained cells. Photos were taken with Zeiss Filter set 1, excitation: band pass (BP) 365/12 nm and emission: long pass (LP) 397 nm. (a,b,e,f) SAG 698-1a; (c,d,g,h) SAG 698-1b; Images were taken with a Zeiss Axiovert 200M (Carl Zeiss, Jena, Germany); scale bars: 20 μm. [file Image_8.TIF]

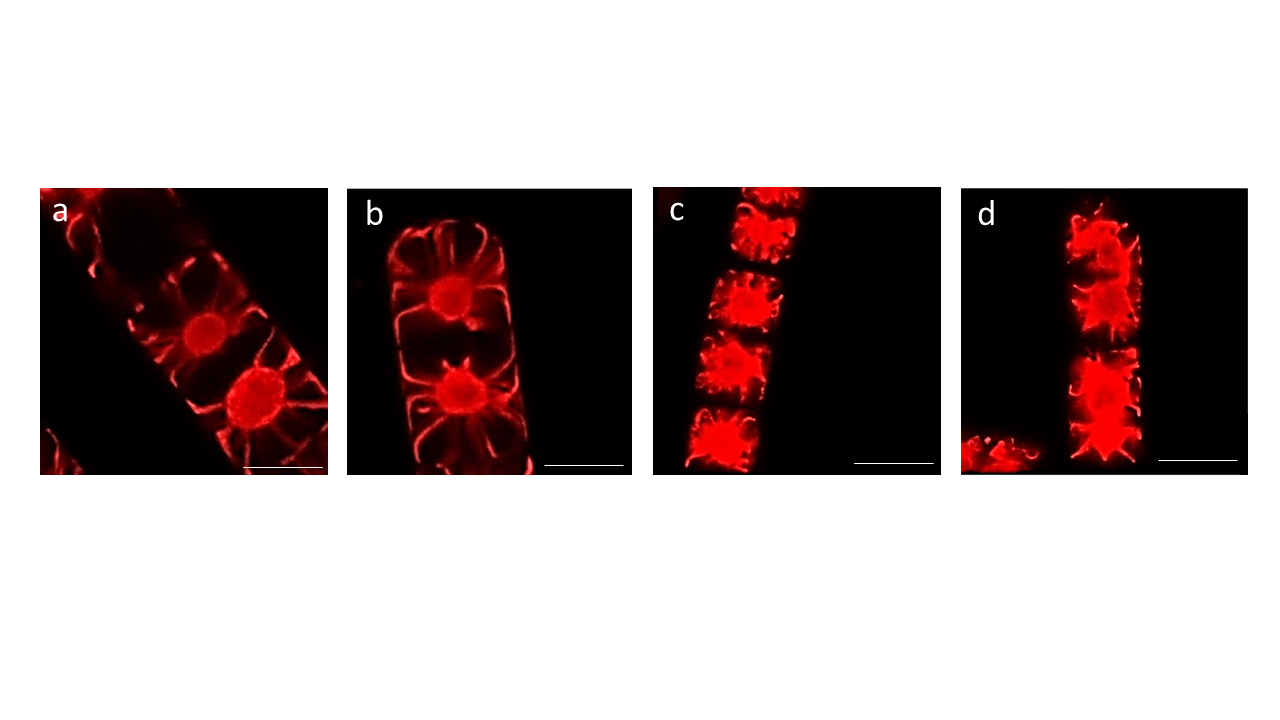

Supplement: Supplementary Figure 9 — Confocal laser scanning images of Zygnema; (a,b) SAG 698-1a; (c,d) SAG 698-1b; Images were taken with a Zeiss Axiovert 200M, Pascal LSM5 (Carl Zeiss, Jena, Germany); scale bars: 20 μm. [file Image_9.TIF]

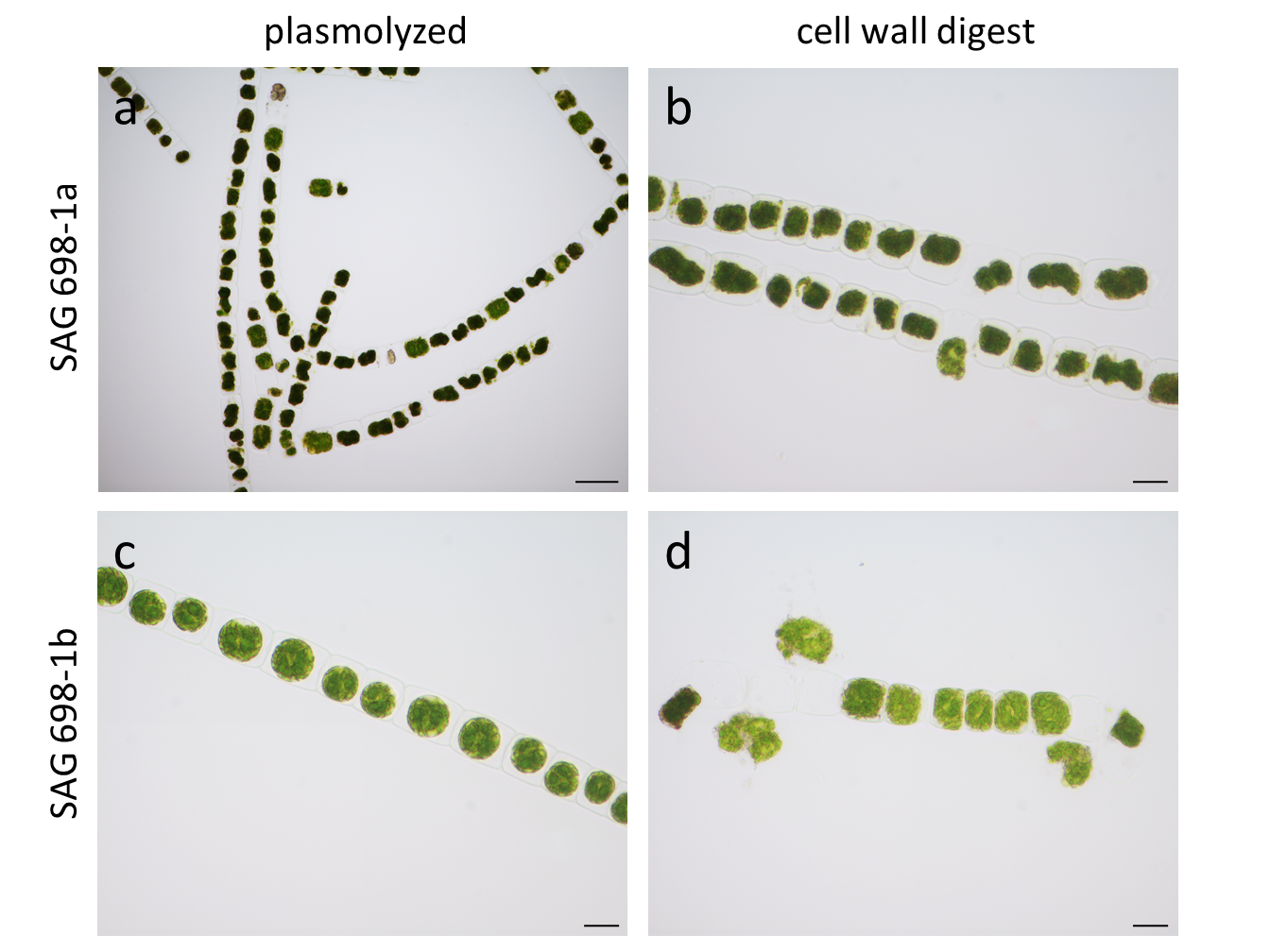

Supplement: Supplementary Figure 10 — Microscope image of SAG 698-1a and SAG 698-1b filaments plasmolyzed and digested with cell wall enzymes. Images were taken under Axio Imager 2 microscope. (a,b) SAG 698-1a; (c,d) SAG 698-1b. Scale bar in (a): 50 μm, in (b–d): 20 μm. [file Image_10.TIF]
